# Longitudinal insights into comorbidity patterns and burden among middle-aged and older adults with diabetes in China: a nine-year cohort study using CHARLS

**DOI:** 10.7189/jogh.15.04353

**Published:** 2025-12-12

**Authors:** Haoqing Tang, Mingyue Li, Huixian Zheng, Yuxun Zhou, Xiaoyun Liu

**Affiliations:** 1 Department of Health Policy and Management, School of Public Health, Peking University, Beijing, China; 2 China Center for Health Development Studies, Peking University, Beijing, China

## Abstract

**Background:**

China has the largest diabetic population, accounting for over a quarter of global cases. As a chronic condition frequently accompanied by comorbidities, diabetes requires research on the patterns and burdens of associated conditions, particularly within primary care settings. We aim to provide longitudinal insights into the evolution of comorbidity patterns and burdens among China’s diabetic population, examining trends and influencing factors.

**Methods:**

We used longitudinal data in five waves (2011–20) of the China Health and Retirement Longitudinal Study (CHARLS). We classified comorbidities into three categories based on their aetiological relationship with diabetes: traditional concordant (TCC), non-traditional concordant (NCC), and discordant (DC). We used generalised estimating equation (GEE) models to identify factors influencing comorbidity burden.

**Results:**

Between 2011 and 2020, the prevalence of diabetes among individuals aged ≥45 years in China increased from 6.3% to 17.6%, while diabetes-related comorbidities rose from 5.3% to 16.9%. The most prevalent comorbidities in 2020 were hypertension (68.3%) and dyslipidemia (62.3%). The average number of conditions per diabetic patient increased by 1.5, and the Charlson comorbidity index (CCI) increased by 50%. At the final follow-up, 65.4% of patients had TCC, 92.4% had NCC, and 75.9% had DC. GEE analysis showed that the number (*β* = 0.034; *P* = 0.009) and CCI (*β* = 0.041; *P* = 0.021) of comorbidities increase with age. Diabetes control was significantly associated with a decrease in both the numbers (*β* = −0.720; *P* < 0.001) and CCI of TCC (*β* = −0.951; *P* < 0.001), a modest but significant reduction in NCC numbers (*β* = −0.134; *P* = 0.021) without a corresponding decrease in its CCI, and no significant association with DC.

**Conclusions:**

The increasing comorbidity burden in diabetic patients highlights the need for primary care-centred interventions tailored to comorbidity types. Targeted management of diabetes is instrumental in reducing the severity of comorbidities within the TCC pattern.

Noncommunicable diseases (NCDs) account for 41 million deaths annually, representing 74% of global mortality [1]. Diabetes, a serious chronic condition, profoundly impacts individuals, families, and societies. In 2021, an estimated 537 million adults aged 20–79 years were living with diabetes, a number projected to rise to 643 million by 2030 and 783 million by 2045 [2]. Globally, diabetes ranks among the top ten causes of death, claiming approximately 6.7 million lives in 2021 [2]. The economic burden is also substantial, with global health expenditures on diabetes reaching approximately USD 966 billion in 2021, a 316% increase over 15 years [3]. China accounts for the largest share of the global diabetes burden, with approximately 141 million adults aged 20–79 years affected in 2021. This represents over a quarter of global cases and is projected to exceed 174 million by 2045 [3]. The prevalence of diabetes in China reached 13.0% in 2021, with average diabetes-related healthcare expenditures of approximately USD 1173.5 per person [2].

Comorbidities – chronic conditions co-occurring with a primary disease – are a significant global public health issue, especially among ageing populations [4–7]. Patients with diabetes are at high risk of multiple comorbidities [8]. Comorbidity is an escalating issue not only in high-income countries but also in low- and middle-income countries (LMICs), where 77% of global deaths from NCDs occur, with 85% classified as premature [1]. Studies reveal that common comorbidities in patients with diabetes include hypertension, lipid disorders, cardiovascular conditions (such as coronary heart disease), microvascular complications, and depression [8]. Compared with patients with only one chronic disease, those with comorbidities face greater threats to safety and quality of life, leading to increased healthcare utilisation, longer hospital stays, and a higher disease burden [9]. This, in turn, affects health expenditures and equitable access to healthcare resources [9]. Some comorbidities are traditional complications of diabetes, while others share common risk factors and pathophysiological pathways. Additionally, some comorbidities are unrelated to diabetes. Specific combinations of comorbidities in patients with diabetes can influence their ability to prioritise and manage their health. For example, diabetes-concordant conditions (those that share management goals with diabetes) generally facilitate higher-quality care. In contrast, discordant conditions (*e.g.* depression and arthritis) can hinder self-management and lifestyle modifications recommended for diabetes [10,11].

To assess the impact of comorbidity, it is essential to measure it accurately. Comorbidity measures fall into two main types: simple disease counts based on patient self-reports or clinical assessments, and indices that assess morbidity burden by weighting various conditions based on factors such as mortality risk, severity, or resource utilisation [12]. The Charlson comorbidity index (CCI) is a widely used tool that captures the presence and severity of diseases, links diagnoses to healthcare resource use, and facilitates comparisons of case complexity across clinical settings, providing insights into the implications of comorbid conditions in practice.

Most international research on comorbidity patterns and burdens relies on cross-sectional data, often focusing on broad interconnections between diseases [13,14]. For example, Prados-Torres and colleagues reviewed 14 articles on comorbidity research and identified 97 combinations of two or more diseases and 63 combinations of three or more diseases [15]. They categorised these into three prominent patterns: cardiometabolic, mental-psychological, and musculoskeletal [15]. Similarly, Zhang and colleagues identified four stable comorbidity patterns among middle-aged and older individuals in China using the China Health and Retirement Longitudinal Study (CHARLS) data from 2011–15: cardiocerebrovascular-metabolic, respiratory, arthritis-organ disease, and mental-memory [16]. While these studies provide a general perspective, they offer limited insights into specific populations such as individuals with diabetes [17]. Diabetes, a highly prevalent chronic condition with significant comorbidity rates, highlights the need for targeted studies examining specific comorbidity patterns and burdens. Most existing studies on comorbidity patterns have been conducted in high-income countries, where epidemiological profiles and healthcare systems differ significantly from those in LMICs [18,19]. However, research specifically examining these patterns and burdens among the diabetic populations in LMICs is limited.

To address this gap, there is an urgent need for longitudinal studies exploring the evolution of comorbidity patterns in diabetic populations within LMICs. Such research is crucial for understanding comorbidity trends and health outcomes in these regions. Considering these gaps, we aimed to provide longitudinal insights into the evolution of comorbidity patterns and burdens in China’s diabetic population. Using a nationally representative sample of middle-aged and older Chinese adults, we focussed on changing trends in comorbidity burden, patterns of comorbidities, and the factors driving these changes. Our findings could offer valuable evidence to inform health policies and interventions tailored to the needs of LMICs.

## METHODS

### Study design

We employed two distinct designs. First, we used a repeated cross-sectional design to analyse the annual prevalence of diabetes, diabetes-related comorbidities, and chronic conditions among individuals with diabetes. Second, we constructed a retrospective cohort study to evaluate long-term trends in comorbidity patterns and burdens, as well as the factors driving these changes.

### Data source

We obtained the data from CHARLS, a continuous national survey targeting Chinese citizens aged ≥45 years across 28 provinces [20]. The CHARLS employs a multistage, stratified, probability-proportional-to-size sampling method to gather a nationally representative sample through one-on-one interviews using a structured questionnaire. The questionnaire covers a wide range of topics, including demographics, health status, healthcare utilisation, health insurance, family dynamics, and income.

The CHARLS baseline survey, conducted between 2011 and 2012, included 17 708 respondents with a response rate of 80.5%. Follow-up surveys were conducted every 2–3 years, and the surveys employed individual weighting variables to maintain national representation. In addition, to address attrition and enhance national representation, new participants were included in each follow-up wave. This design allows CHARLS to be used for both repeated cross-sectional analyses and cohort-based studies, enabling diverse research designs. We used data from five waves of CHARLS conducted in 2011, 2013, 2015, 2018, and 2020. A detailed description of CHARLS objectives and methodology is available elsewhere [20].

The primary outcome variables were comorbidity patterns and burdens. In this study, we defined comorbidity as the coexistence of diabetes with one or more chronic NCDs [6,21]. We used 14 self-reported chronic NCDs to measure comorbidities in diabetes, including diabetes, hypertension, dyslipidemia, arthritis or rheumatism, heart disease, gastrointestinal or digestive system disease, chronic lung disease, kidney disease, stroke, liver disease, memory-related disorders, asthma, emotional and mental disorders, and cancer. We defined diabetes as a self-reported diagnosis by a doctor or the self-reported use of antidiabetic medications. We assessed this with two questions: ‘Have you been diagnosed with diabetes or high blood glucose by a doctor?’ and ‘Are you now currently using any treatments for diabetes or its complications, such as Chinese traditional medicine, modern Western medicine, or other treatments?’. We calculated the number of chronic NCDs (excluding diabetes) for each participant to determine the total number of comorbidities.

We employed CCI, a widely used tool, to measure comorbidity burden by assigning weighted scores to various chronic conditions based on mortality risk, severity, or likely resource utilisation [22]. The weighting system was based on the original CCI framework (Table S1 in the **Online Supplementary Document**), informed by subsequent updates from Charlson (2008) and Quan (2011), and refined in accordance with Glasheen and colleagues (2019), which incorporated explicit weights for additional chronic conditions including hypertension and rheumatism [23–25].

We categorised the comorbidities into three distinct patterns based on their aetiological relationship with diabetes [8,26]:

1. Traditional concordant comorbidity (TCC): These include the traditional complications of diabetes that align with its core pathophysiological profile and management focus. Individuals with heart disease, stroke, or kidney disease were categorised as having TCC.
2. Non-traditional concordant comorbidity (NCC): This category includes conditions that share common risk factors and pathophysiological pathways with diabetes. Although these conditions are associated with diabetes, their connection is generally weaker, and they are not traditionally considered diabetic complications due to their lower specificity and degree of association. Patients with hypertension, dyslipidemia, cancer, memory-related disorders, and liver disease were classified under the NCC.
3. Discordant comorbidity (DC): These are conditions with no clear aetiologic link to diabetes, but that frequently co-occur with it, significantly impacting patient mortality and quality of life. Patients with gastrointestinal or digestive system disease, chronic lung disease, asthma, emotional and mental disorders, arthritis, or rheumatism were classified as having DC.

As individuals may have multiple comorbidities across these categories, the sum of the categories may exceed 100%.

### Statistical analysis

We analysed trends in comorbidity among individuals with diabetes using data from five CHARLS waves (2011, 2013, 2015, 2018, and 2020). We initially reported the prevalence of diabetes, diabetes-related comorbidities, and chronic conditions among individuals with diabetes in each wave.

Subsequently, we constructed a retrospective cohort to examine long-term trends in comorbidity patterns and burdens. Based on individuals entering the cohort, we considered the first wave, in which participants self-reported having diabetes, the baseline (F0), while F1–4 represented subsequent follow-up waves. As participants entered the cohort at different times, we excluded individuals without diabetes and those lost to follow-up after the initial survey to ensure consistency in the trend analysis.

We used descriptive analyses to report the characteristics of the study sample, the prevalence of diabetes-related comorbidities, and long-term trends in comorbidity patterns and burdens.

To identify factors influencing long-term trends in comorbidity patterns and burdens within the diabetic cohort, we adopted a generalised estimating equation regression for longitudinal data. This approach accounts for within-subject correlations across repeated measures and provides robust estimates of associations between covariates and outcomes across follow-up waves.

We specified the model with an exchangeable correlation structure. All analyses accounted for the complex survey design of CHARLS by applying individual sampling weights to ensure national representativeness. We included time-varying covariates, including age, socioeconomic status, health insurance type, and diabetes control status, as wave-specific updated variables corresponding to each survey round. To address potential attrition and survival bias due to loss to follow-up, we applied inverse probability-of-censoring weighting in the longitudinal analyses [27].

We included variables that may influence the burden of diabetes-related comorbidities, including age (45–54, 55–64, 65–74, and ≥75 years), sex (male and female), marital status (married or partnered, unmarried, and others), education level (less than lower secondary, upper secondary and vocational training, college and above), residence status (rural, urban, and rural-to-urban, defined as individuals with agricultural household registration but residing in urban areas), health insurance type (none, Urban Employee Basic Medical Insurance, Urban and Rural Resident Basic Medical Insurance, and others), socioeconomic group (quintiles from lowest to highest based on per capita household consumption expenditure), and diabetes control status.

We measured diabetes control from the self-reported question: ‘Is your blood glucose generally under control?’. To improve measurement accuracy, we further cross-validated the self-reported responses against biomarker data from the 2011 and 2015 waves, during which blood samples were collected. Specifically, we used fasting HbA1c values to correct self-reported control status, defining glycaemic control as HbA1c <7.0% for participants aged 45–64 years and HbA1c <8.5% for those aged ≥65 years, in accordance with clinical recommendations for older adults [28].

We used STATA, version 17.0 (Stata Corp., College Station, Texas, USA) for all analyses, with a significance level set at *P* < 0.05.

## RESULTS

### Descriptive statistics

The mean (x̄) age of participants increased from 2011 (x̄ = 59.1; standard deviation (SD) = 9.8) to 2020 (x̄ = 61.8; SD = 9.9) (**Table 1**). Gender distribution remained relatively balanced, with a slight female majority in each wave (51.2–52.7%). Around 87.0% of participants had educational attainment below the lower junior high school level. Rural residency was predominant, accounting for 58.7–61.3% of the sample, while approximately 20% were rural-to-urban migrants. Health insurance coverage was high, with 91.3–97.0% of participants enrolled in at least one insurance programme.

**Table 1:** Demographic characteristics of study population in China, 2011–20*

	2011 (n = 16 931)	2013 (n = 17 974)	2015 (n = 19 719)	2018 (n = 19 454)	2020 (n = 19 157)
**Age**					
45–54	6119 (36.1)	5973 (33.2)	6812 (34.5)	5744 (29.5)	5312 (27.7)
55–64	6299 (37.2)	6667 (37.1)	6648 (33.7)	6316 (32.5)	6524 (34.1)
65–74	3093 (18.3)	3616 (20.1)	4303 (21.8)	5013 (25.8)	5119 (26.7)
≥75	1420 (8.4)	1718 (9.6)	1956 (9.9)	2381 (12.2)	2202 (11.5)
**Sex**					
Male	8268 (48.8)	8732 (48.6)	9605 (48.7)	9283 (47.7)	9062 (47.3)
Female	8663 (51.2)	9242 (51.4)	10114 (51.3)	10171 (52.3)	10095 (52.7)
**Marital status**					
Married/partnered	14724 (87.0)	15619 (86.9)	17046 (86.4)	16549 (85.1)	16032 (83.7)
Unmarried/other	2197 (13.0)	2352 (13.1)	2673 (13.6)	2905 (14.9)	3125 (16.3)
**Education**					
Less than lower secondary	14848 (87.7)	15731 (87.5)	17311 (87.8)	16994 (87.4)	15334 (87.8)
Upper secondary and vocational training	1732 (10.2)	1886 (10.5)	1969 (10.0)	2046 (10.5)	1796 (10.3)
College and above	345 (2.0)	355 (2.0)	433 (2.2)	414 (2.1)	335 (1.9)
**Residence status**					
Rural	9716 (58.7)	10208 (58.6)	9995 (58.4)	10237 (59.2)	11058 (61.3)
Urban	3471 (21.0)	3738 (21.5)	3510 (20.5)	3534 (20.5)	3796 (21.0)
Rural-to-urban	3363 (20.3)	3479 (20.0)	3597 (21.0)	3507 (20.3)	3186 (17.7)
**Health insurance**					
None	1104 (6.6)	688 (3.9)	1648 (8.7)	581 (3.0)	914 (4.8)
UEBMI	1724 (10.3)	2093 (11.8)	1808 (9.5)	2528 (13.0)	2700 (14.1)
URRBMI	12980 (77.7)	13984 (79.2)	14035 (74.0)	15035 (77.5)	15092 (78.8)
Others	900 (5.4)	900 (5.1)	1477 (7.8)	1266 (6.5)	450 (2.3)
**Socioeconomic group**					
Quintile 1 (lowest)	2823 (20.0)	2355 (20.0)	2655 (20.0)	3237 (20.0)	2807 (19.6)
Quintile 2	2820 (20.0)	2352 (20.0)	2643 (20.0)	3236 (20.0)	2878 (20.1)
Quintile 3	2825 (20.0)	2353 (20.0)	2650 (20.0)	3237 (20.0)	2894 (20.2)
Quintile 4	2818 (20.0)	2354 (20.0)	2648 (20.0)	3236 (20.0)	2896 (20.2)
Quintile 5 (highest)	2821 (20.0)	2352 (20.0)	2649 (20.0)	3236 (20.0)	2827 (19.8)

UEBMI – Urban Employee Basic Medical Insurance, URRBMI – Urban and Rural Resident Basic Medical Insurance

*Values are presented as numbers (percentages).

### Prevalence of diabetes and comorbidity

Among adults aged ≥45 years in China, the prevalence of diabetes increased from 6.3% in 2011 to 17.6% in 2020 (Table S2 in the **Online Supplementary Document**). Across all survey waves, the prevalence of diabetes was higher in urban areas than in rural areas. In 2011, the prevalence in urban areas was 11.1%, compared to 4.5% in rural areas. By 2020, the prevalence was 22.0% in urban and 15.8% in rural areas. Similarly, diabetes prevalence rose across socioeconomic groups: from 4.8% in the lowest quintile and 9.0% in the highest quintile in 2011, to 15.9% in the lowest quintile and 19.9% in the highest quintile in 2020. Overall, the prevalence of comorbidities in the study population increased significantly, from 5.3% in 2011 to 16.9% in 2020. Comorbidity prevalence rose with age, reaching its highest rate (21.8%) in the 65-74 age group in 2020. In 2020, urban residents exhibited a higher prevalence of comorbidity (21.2%) than rural residents (15.2%) and rural-to-urban migrants (17.4%). The prevalence also increased with socioeconomic status, with the highest rates observed in the top quintile (19.3%) compared to the lowest quintile (15.5%) in 2020.

**Figure 1:**
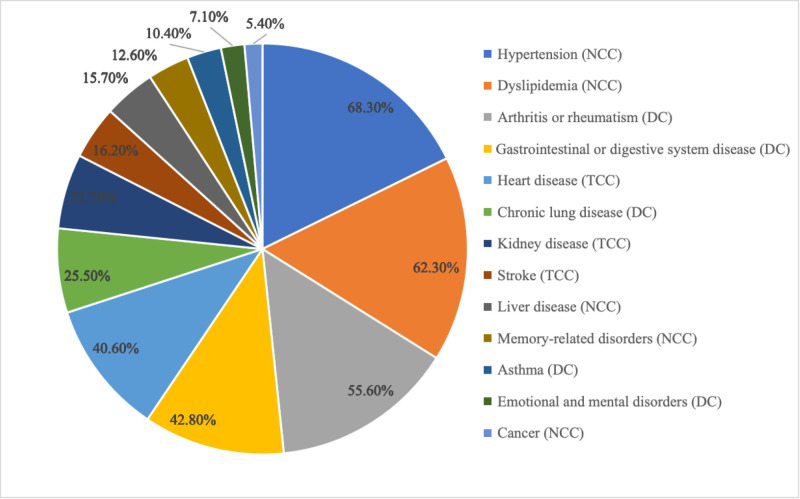
Prevalence of chronic conditions among people with diabetes in China, 2020.

Furthermore, the most common comorbidities were hypertension (68.3%), dyslipidemia (62.3%), and arthritis or rheumatism (55.6%) (**Figure 1**). Gastrointestinal or digestive system diseases (42.8%) and heart diseases (40.6%) were also relatively prevalent, while cancer was rare (5.4%).

### Trends in comorbidity burden and patterns

At baseline (F0), 89.0% of participants with diabetes had comorbid conditions, with an average of 2.7 comorbidities (SD = 1.9) and a mean CCI score of 3.0 (SD = 2.4) (Table S3 in the **Online Supplementary Document**). In the subsequent follow-up waves (F1–4), both the percentage of participants with comorbidities and the average number of comorbidities increased. By the final follow-up (F4), 96.7% of the participants reported comorbid conditions, with an average of 4.2 comorbidities (SD = 2.3) and a mean CCI score of 5.0 (SD = 3.3). All three comorbidity categories increased over time. From baseline (F0) to the final follow-up (F4), TCCs (46.7% to 65.4%), NCCs (80.6% to 92.4%), and DCs (66.9% to 75.9%) all showed growth, with the most pronounced increase observed in NCCs.

From F0 to F4, the average number of conditions increased across all three comorbidity categories: TCCs from 0.6 to 1.0, NCCs from 1.3 to 1.9, and DCs from 1.1 to 1.5.

### Factors influencing comorbidity burden among people with diabetes

Older age was positively correlated with comorbidity count (*β* = 0.034; *P* = 0.009) (**Table 2**). Rural-to-urban migrants (*β* = −0.477; *P* = 0.030) showed a significantly negative association with comorbidity counts compared with rural residents. Education level, health insurance type, socioeconomic quintiles and diabetes control showed no significant association with the number of comorbidities, indicating that these factors have a limited influence on the comorbidity count. Across the three comorbidity patterns, diabetes control showed varying associations with the onset of different types of comorbidities. This was strongly linked to a significant reduction in TCC (*β* = −0.720; *P* < 0.001), while it was modestly but significantly associated with a decrease in NCC (*β* = −0.134; *P* = 0.021). However, no significant association was observed with the number of DCs.

**Table 2:** Multivariate regression analysis of factors influencing the increase in the numbers of comorbidities among people with diabetes in China

	Total numbers		TCC numbers		NCC numbers		DC numbers	
	***β* (95% CI)**	***P*-value**	***β* (95% CI)**	***P*-value**	***β* (95% CI)**	***P*-value**	***β* (95% CI)**	***P*-value**
**Age**	0.034 (0.009, 0.060)	<0.01	0.004 (–0.003, 0.011)		–0.001 (–0.012, 0.009)		0.018 (–0.000, 0.037)	
**Sex**								
Male	ref		ref		ref		ref	
Female	0.237 (−0.107, 0.582)		–0.002 (–0.117, 0.113)		0.095 (–0.053, 0.242)		–0.097 (–0.336, 0.142)	
**Education**								
Less than lower secondary	ref		ref		ref		ref	
Upper secondary and vocational training	–0.399 (–0.858, 0.060)		–0.006 (–0.178, 0.165)		0.017 (–0.193, 0.227)		–0.306 (–0.606, –0.007)	<0.05
College and above	–0.415 (–1.291, 0.461)		–0.039 (–0.326, 0.248)		–0.213 (–0.559, 0.133)		–0.294 (–1.135, 0.546)	
**Residence status**								
Rural	ref		ref		ref		ref	
Urban	0.097 (–0.367, 0.561)		0.085 (–0.085, 0.256)		0.252 (0.034, 0.469)		–0.225 (–0.496, 0.047)	
Rural-to-urban	–0.477 (–0.907, –0.047)	<0.05	–0.111 (–0.246, 0.025)		–0.042 (–0.217, 0.133)		–0.148 (–0.410, 0.114)	
**Health insurance**								
None	ref		ref		ref		ref	
UEBMI	–0.100 (–0.520, 0.319)		–0.129 (–0.387, 0.129)		–0.061 (–0.295, 0.172)		–0.124 (–0.371, 0.122)	
URRBMI	0.004 (–0.349, 0.356)		–0.076 (–0.323, 0.170)		0.142 (–0.039, 0.322)		–0.182 (–0.406, 0.041)	
Others	–0.011 (–0.446, 0.425)		0.018 (–0.307, 0.343)		–0.064 (–0.308, 0.180)		0.176 (–0.164, 0.517)	
**Socioeconomic group**								
Quintile 1 (lowest)	ref		ref		ref		ref	
Quintile 2	0.126 (–0.102, 0.354)		0.050 (–0.084, 0.183)		0.084 (–0.062, 0.230)		0.080 (–0.104, 0.265)	
Quintile 3	0.151 (–0.104, 0.405)		0.084 (–0.056, 0.224)		–0.042 (–0.186, 0.103)		0.137 (–0.049, 0.324)	
Quintile 4	0.211 (–0.068, 0.490)		0.111 (–0.051, 0.272)		0.050 (–0.114, 0.213)		0.152 (–0.034, 0.338)	
Quintile 5 (highest)	0.096 (–0.181, 0.374)		0.028 (–0.114, 0.170)		0.052 (–0.114, 0.218)		0.100 (–0.082, 0.282)	
**Diabetes control**								
No	ref		ref		ref		ref	
Yes	–0.120 (–0.340, 0.099)		–0.720 (–0.850, –0.590)	<0.001	–0.134 (–0.247, –0.020)	<0.05	–0.069 (–0.222, 0.084)	

CI – confidence interval, ref – reference, UEBMI – Urban Employee Basic Medical Insurance, URRBMI – Urban and Rural Resident Basic Medical Insurance

Age was positively associated with an increase in the CII of comorbidities association, meaning that older individuals tended to have a higher comorbidity burden (*β* = 0.041; *P* = 0.021) (**Table 3**). Rural-to-urban migrants (*β* = −0.634; *P* = 0.030) showed a significantly negative association with CCI compared with rural residents. Individuals with upper secondary or vocational education (*β* = –0.783; *P* = 0.011) had a significantly lower CCI score compared with those with less than lower secondary education. Health insurance type, socioeconomic quintiles and diabetes control showed no significant associations with the number of comorbidities, indicating that these factors have a limited influence on CCI. Across the three comorbidity patterns, diabetes control showed varying associations with CCI. This was strongly linked to a significant reduction in the TCC CCI (*β* = −0.951; *P* < 0.001). Additionally, no significant association was found between the NCC CCI and DC CCI.

**Table 3:** Multivariate regression analysis of factors influencing the increase in the CCI of comorbidities among people with diabetes in China

	Total numbers		TCC numbers		NCC numbers		DC numbers	
	***β* (95% CI)**	***P*-value**	***β* (95% CI)**	***P*-value**	***β* (95% CI)**	***P*-value**	***β* (95% CI)**	***P*-value**
**Age**	0.041 (0.006, 0.076)	<0.05	0.002 ()	−0.010, 0.015	0.005 ()	−0.021, 0.030	0.018	−0.000, 0.037
**Sex**								
Male	ref		ref		ref		ref	
Female	0.298 (−0.177, 0.772)		−0.144 ()	−0.335, 0.047	0.285 (−0.080, 0.651)		−0.097 (−0.336, 0.142)	
**Education**								
Less than lower secondary	ref		ref		ref		ref	
Upper secondary and vocational training	−0.783 (−1.383, −0.183)	<0.05	−0.171 (−0.440, 0.098)		−0.303 (−0.751, 0.145)		−0.306 (−0.606, −0.007)	<0.05
College and above	−0.508 (−1.602, 0.585)		−0.125 (−0.589, 0.339)		−0.372 (−1.296, 0.553)		−0.294 (−1.135, 0.546)	
**Residence status**								
Rural	ref		ref		ref		ref	
Urban	0.371 (−0.337, 1.078)		0.058 (−0.244, 0.360)		0.644 (0.064, 1.225)	<0.05	−0.225 (−0.496, 0.047)	
Rural-to-urban	−0.634 (−1.206, −0.061)	<0.05	−0.225 (−0.459, 0.008)		−0.126 (−0.505, 0.253)		−0.148 (−0.410, 0.114)	
**Health insurance**								
None	ref		ref		ref		ref	
UEBMI	−0.129 (−0.692, 0.434)		−0.143 (−0.505, 0.218)		−0.202 (−0.660, 0.256)		−0.124 (−0.371, 0.122)	
URRBMI	0.029 (−0.435, 0.493)		−0.082 (−0.428, 0.264)		0.154 (−0.160, 0.468)		−0.182 (−0.406, 0.041)	
Others	−0.228 (−0.847, 0.391)		0.052 (−0.442, 0.546)		−0.397 (−0.969, 0.174)		0.176 (−0.164, 0.517)	
**Socioeconomic group**								
Quintile 1 (lowest)	ref		ref		ref		ref	
Quintile 2	0.164 (−0.150, 0.478)		−0.019 (−0.231, 0.193)		0.143 (−0.127, 0.412)		0.080 (−0.104, 0.265)	
Quintile 3	0.153 (−0.183, 0.488)		0.038 (−0.162, 0.237)		0.014 (−0.251, 0.278)		0.137 (−0.049, 0.324)	
Quintile 4	0.267 (−0.158, 0.692)		0.094 (−0.143, 0.330)		0.163 (−0.229, 0.556)		0.152 (−0.034, 0.338)	
Quintile 5 (highest)	0.216 (−0.209, 0.641)		0.033 (−0.179, 0.245)		0.210 (−0.182, 0.602)		0.100 (−0.082, 0.282)	
**Diabetes control**								
No	ref		ref		ref		ref	
Yes	−0.173 (− 0.464, 0.117)		−0.951 (−1.148, −0.754)	<0.001	−0.038 (−0.263, 0.187)		−0.069 (−0.222, 0.084)	

CI – confidence interval, ref – reference, UEBMI – Urban Employee Basic Medical Insurance, URRBMI – Urban and Rural Resident Basic Medical Insurance

## DISCUSSION

As one of the most populous countries in the world, China faces a growing diabetes epidemic that affects millions of individuals, placing immense pressure on public health systems and economic resources [29–31]. We employed a cohort approach to examine the evolving patterns and burden of diabetes-related comorbidities over time, using a nationally representative sample of middle-aged and older Chinese adults. By tracking these trends, we provide a longitudinal perspective on how comorbidity dynamics shift as diabetes progresses. First, we found that diabetes-related comorbidities rose from 5.3% in 2011 to 16.9% in 2020. This surge is partly attributed to population growth, urbanisation, ageing, rising obesity rates, and sedentary lifestyles. These factors contributed to an increase in the diabetic population from 98.4 million in 2013 to 140.9 million in 2021 [3,32–34].

Compared to patients newly diagnosed with elevated blood glucose levels, those with a longer history of diabetes tend to experience a greater number and diversity of comorbidities [35]. This elevated risk reflects the cumulative impact of prolonged exposure to hyperglycaemia and insulin resistance, which can damage multiple organ systems, particularly through effects on the microvasculature, macrovasculature, and immune response. Among individuals with diabetes, these effects roughly double the risk of myocardial infarction, increase the risk of renal failure by 5-fold, and lead to over a 10-fold increase in the likelihood of amputation and blindness [36]. However, the growing prevalence of diabetes-related comorbidities is also influenced by longer life expectancies and declining mortality rates, resulting in a broader range of complications and causes of death [37]. Individuals with multiple chronic conditions often encounter barriers to self-care, including physical limitations, limited knowledge, financial constraints, difficulties accessing care, and the need for social and emotional support [37,38]. This study highlights the rapid increase in both the number and severity (measured by CCI scores) of comorbidities among patients with diabetes. By the final follow-up, the average number of conditions per patient with diabetes increased by 1.5 (from 2.7 to 4.2), and the comorbidity burden increased by 50%, from 3.0 to 5.0.

Second, we revealed that among the five most recently identified comorbidities, only heart disease falls within the TCC category. Heart disease aligns with the well-established pathophysiological effects of diabetes on cardiovascular health, which are extensively documented in academic literature and clinical guidelines [39,40]. This association is rooted in shared risk factors such as hyperglycaemia, insulin resistance, and inflammation, which are central to cardiovascular pathology in individuals with diabetes [2,41]. However, the other four common comorbidities presented a different narrative. Hypertension and dyslipidemia, though sharing metabolic risk factors with diabetes, such as obesity and a sedentary lifestyle, are not directly considered diabetes-specific complications, but are instead seen as part of the broader metabolic syndrome affecting these patients [8,42]. The prevalence and impact of these NCC challenge the conventional focus solely on hyperglycaemia in diabetes management, suggesting the need for a more integrative approach to treatment that addresses multiple interlinked risk factors [41]. Arthritis, rheumatism, and gastrointestinal diseases represent DC that lack a direct pathophysiological link to diabetes. Often overlooked in management guidelines, their prevalence in patients with diabetes indicates the possible systemic effects of diabetes or shared, yet poorly understood, aetiological factors [39,40]. These findings underscore the need for a broader investigation into how diabetes may indirectly influence other organ systems. At present, there is no universally accepted framework for categorising diabetes-related comorbidities, and existing classifications remain largely exploratory. Future studies incorporating biomarker data, longitudinal follow-up, and broader clinical validation are needed to further refine and validate this classification framework.

Third, we focused on factors influencing the changing trend of comorbidity burden across different patterns of comorbidity. Our analysis highlighted the complex relationship between diabetes control and the burden of various comorbidity patterns. Importantly, diabetes management showed different associations across specific comorbidity patterns. The significant negative association between diabetes control and TCC burden (both count and CCI) underscores the critical role of maintaining optimal glycaemic levels to mitigate well-established complications, suggesting that targeted diabetes management is instrumental in reducing the severity of comorbidities within the TCC pattern. A previous study found that chronic hyperglycaemia is associated with microvascular and macrovascular complications, reduced quality of life and premature mortality [43]. In the UK Prospective Diabetes Study, the incidence of complications was significantly associated with blood glucose levels. Each 10.9 mmol/mol (1%) reduction in the updated mean HbA1c was linked to a 21% reduction in diabetes-related deaths and a 37% reduction in microvascular complications [44]. Systematic reviews and meta-analyses have also demonstrated a positive relationship between HbA1c and the risk of macrovascular outcomes and mortality [45]. The importance of early glycaemic control was first highlighted in the UK study, where tight control initiated at the diagnosis of type 2 diabetes reduced the risk of myocardial infarction and mortality by 19–20% after ten years. In contrast, delaying glycaemic control reduced these risks by only 3% and 6.5%, respectively [46].

In contrast, diabetes control was modestly but significantly associated with a decrease in NCC count but was not significantly related to changes in the CCI. This suggests that diabetes management may still provide preventive benefits, particularly by mitigating early-stage risks. These findings suggest that maintaining reasonable glucose control may be associated with a lower risk of developing certain comorbidities, even those less directly related to diabetes [43].

However, no significant association was observed between diabetes control and DC reduction. This lack of association highlights the limitations of glycaemic management in addressing comorbid conditions that do not share the underlying risk factors or biological pathways with diabetes. The absence of a significant impact on DC highlights the need for alternative, non-glycaemic-focussed strategies to manage discordant health conditions. This finding emphasises the importance of comprehensive healthcare approaches that address a wide range of patient needs beyond diabetes management alone [43].

This study has several limitations. First, the use of self-reported measures of chronic diseases may have led to underestimation of their prevalence, particularly among older adults and those with lower socioeconomic or educational status who are more prone to underreporting. Although the self-reported diabetes control variable was cross-validated using biomarker data from the 2011 and 2015 waves, this correction could not be applied to other years because blood samples were unavailable, which may affect the consistency of this measure. Second, the CHARLS database does not distinguish between type 1 and type 2 diabetes, and its broad disease categories and lack of detailed International Classification of Diseases-10 categories may have limited the precision of disease identification and reduced analytical specificity. Moreover, the exact time of diabetes diagnosis was unavailable, preventing assessment of disease duration. Third, due to data limitations, we could not control some contextual and time-varying confounders – such as policy changes, regional healthcare access, obesity, diet, medication use, and diabetes duration – which may have introduced residual confounding.

## CONCLUSIONS

The comorbidity rates among individuals aged ≥45 years with diabetes in China continued to rise from 2011 to 2020, with the burden of these comorbidities increasing with disease duration. The benefits of diabetes control are most pronounced for comorbidities directly linked to diabetes through shared pathophysiological mechanisms, such as TCC. While diabetes control is associated with lower levels of NCC burden, this association appears much weaker for DC, underscoring the need for a differentiated approach to managing comorbidities among patients with diabetes.

Primary care-centred tailored interventions that address both glycaemic control and broader systemic health strategies are essential to optimise outcomes in this population. Future studies should explore the potential pathways underlying the associations between diabetes and DC, leveraging detailed biomarkers and advanced imaging to elucidate possible systemic mechanisms. Longitudinal data with granular International Classification of Diseases-10 categories and differentiation between diabetes types could improve specificity. Additionally, evaluating the efficacy of integrative management strategies targeting glycaemic and non-glycaemic pathways may optimise care for patients with complex comorbidity profiles.

## Additional material


Online Supplementary Document
