# Supplementary material for: Longitudinal insights into comorbidity patterns and burden among middle-aged and older adults with diabetes in China: a nine-year cohort study using CHARLS
Source: J Glob Health. 2025 Dec 12;15:04353. doi: 10.7189/jogh.15.04353 (PMC12699500; doi:10.7189/jogh.15.04353)
Supplement: Online Supplementary Document [file jogh-15-04353-s001.pdf]

Supplement to: Tang H, Li M, Zheng H, Zhou Y, Liu X. Longitudinal insights into comorbidity patterns and burden among middle-aged and older adults with diabetes in China: a 9-year cohort study using CHARLS. J Glob Health. 2025;15:04353.

Table S1. Weighted index of comorbidity [24,25]

**Condition**	**Weight**
Diabetes	1
Hypertension	1
Dyslipidemia	1
Arthritis or rheumatism	1
Heart disease	1
Gastrointestinal or digestive system disease	1
Chronic lung disease	1
Kidney disease	2
Stroke	1
Liver disease	2
Memory-related disorders	1
Asthma	1
Emotional and mental disorders	1
Cancer	6

Table S2. The prevalence of diabetes and of individuals with both diabetes and at least one comorbidity among the total study population in China, 2011–20 [n(%)]

Variables	2011 wave		2013 wave		2015 wave		2018 wave		2020 wave	
	Diabetes	Comorbidity	Diabetes	Comorbidity	Diabetes	Comorbidity	Diabetes	Comorbidity	Diabetes	Comorbidity
Total	1042 (6.3%)	891 (5.3%)	1293 (7.6%)	1135 (6.3%)	1656 (10.0%)	1522 (7.7%)	2769 (14.2%)	2623 (13.5%)	3362 (17.6%)	3229 (16.9%)

Age (years)										
45-54	269 (4.5%)	215 (3.5%)	287 (5.2%)	236 (4.0%)	340 (7.0%)	298 (4.4%)	517 (9.0%)	460 (8.0%)	605 (11.4%)	557 (10.5%)
55-64	467 (7.5%)	395 (6.3%)	553 (8.8%)	488 (7.3%)	635 (10.7%)	582 (8.8%)	942 (14.9%)	885 (14.0%)	1205 (18.5%)	1152 (17.7%)
65-74	224 (7.3%)	204 (6.6%)	340 (9.7%)	308 (8.5%)	502 (12.7%)	473 (11.0%)	931 (18.6%)	910 (18.2%)	1136 (22.2%)	1116 (21.8%)
75 and above	82 (5.9%)	77 (5.4%)	113 (7.0%)	103 (6.0%)	179 (10.0%)	169 (8.6%)	379 (15.9%)	368 (15.5%)	416 (19.0%)	404 (18.3%)
Sex										
Male	456 (5.6%)	384 (4.6%)	566 (6.9%)	486 (5.6%)	696 (8.8%)	626 (6.5%)	1195 (12.9%)	1113 (12.0%)	1456 (16.1%)	1385 (15.3%)
Female	586 (6.9%)	507 (5.9%)	727 (8.3%)	649 (7.0%)	960 (11.1%)	896 (8.9%)	1574 (15.5%)	1510 (14.8%)	1906 (18.9%)	1844 (18.3%)
Marital status										
Married or Partnered	915 (6.3%)	781 (5.3%)	1117 (7.6%)	975 (6.2%)	1411 (9.9%)	1294 (7.6%)	2318 (14.0%)	2192 (13.2%)	2753 (17.2%)	2640 (16.5%)
Unmarried and Others	126 (5.9%)	109 (5.0%)	176 (7.9%)	160 (6.8%)	245 (10.3%)	228 (8.5%)	451 (15.5%)	431 (14.8%)	609 (19.5%)	589 (18.8%)
Education										
Less than lower secondary	879 (6.0%)	754 (5.1%)	1093 (7.3%)	961 (6.1%)	1421 (9.7%)	1307 (7.6%)	2371 (14.0%)	2257 (13.3%)	2695 (17.6%)	2600 (17.0%)
Upper secondary and vocational training	121 (7.1%)	100 (5.8%)	156 (9.0%)	133 (7.1%)	180 (11.2%)	163 (8.3%)	313 (15.3%)	289 (14.1%)	349 (19.4%)	330 (18.4%)
College and above	40 (11.9%)	35 (10.1%)	44 (13.6%)	41 (11.5%)	55 (16.7%)	52 (12.0%)	85 (20.5%)	77 (18.6%)	76 (22.8%)	71 (21.2%)
Residence status										
Rural	427 (4.5%)	363 (3.7%)	540 (5.5%)	476 (4.7%)	720 (7.9%)	664 (6.6%)	1284 (12.5%)	1227 (12.0%)	1741 (15.8%)	1676 (15.2%)
Urban	379 (11.1%)	333 (9.6%)	448 (13.1%)	405 (10.8%)	472 (15.8%)	440 (12.5%)	722 (20.5%)	687 (19.4%)	831 (22.0%)	806 (21.2%)
Rural-to-urban	214 (6.5%)	177 (5.3%)	263 (8.0%)	219 (6.3%)	318 (10.0%)	283 (7.9%)	517 (14.8%)	477 (13.6%)	587 (18.5%)	555 (17.4%)
Health insurance										
None	57 (5.3%)	48 (4.3%)	34 (5.2%)	29 (4.2%)	119 (8.7%)	111 (6.7%)	50 (8.6%)	45 (7.7%)	155 (17.0%)	147 (16.1%)
UEBMI	207 (12.1%)	180 (10.4%)	256 (13.2%)	232 (11.1%)	223 (15.6%)	207 (11.4%)	507 (20.1%)	483 (19.1%)	610 (22.6%)	584 (21.6%)

URRBMI	691 (5.4%)	588 (4.5%)	894 (6.7%)	784 (5.6%)	1093 (9.1%)	1003 (7.1%)	1991 (13.2%)	1893 (12.6%)	2510 (16.7%)	2413 (16.0%)
Others	82 (9.2%)	70 (7.8%)	98 (11.7%)	80 (8.9%)	156 (13.5%)	143 (9.7%)	217 (17.2%)	198 (15.6%)	87 (19.4%)	85 (18.9%)
Socioeconomic group										
Quintile1 (lowest)	135 (4.8%)	105 (3.7%)	147 (6.5%)	130 (5.5%)	197 (8.3%)	182 (6.9%)	435 (13.4%)	416 (12.9%)	446 (15.9%)	434 (15.5%)
Quintile2	138 (4.9%)	120 (4.3%)	154 (6.8%)	133 (5.7%)	201 (8.8%)	181 (6.8%)	433 (13.4%)	406 (12.5%)	478 (16.6%)	460 (16.0%)
Quintile3	147 (5.2%)	125 (4.4%)	164 (7.3%)	140 (5.9%)	203 (9.3%)	186 (7.0%)	448 (13.8%)	419 (12.9%)	497 (17.2%)	472 (16.3%)
Quintile4	194 (6.9%)	170 (6.0%)	188 (8.4%)	174 (7.4%)	259 (11.9%)	241 (9.1%)	495 (15.3%)	474 (14.6%)	522 (18.0%)	506 (17.5%)
Quintile5 (highest)	251 (9.0%)	219 (7.8%)	215 (9.7%)	180 (7.7%)	265 (12.5%)	247 (9.3%)	512 (15.8%)	479 (14.8%)	561 (19.9%)	545 (19.3%)

Note: Percentages represent the prevalence of individuals with diabetes and those with diabetes combined with ≥1 comorbidities within the total study population, rather than within the diabetic subgroup.

Table S3. Trends in burden and pattern of comorbidities among people with diabetes in China over follow-up waves

	**Diabetes [n]**	**Comorbidity [n(%)]**	**Burden**		**Pattern**					
					**TCC**		**NCC**		**DC**	
			**Numbers of comorbidities [mean(sd)]**	**CCI of comorbidities [mean(sd)]**	**Percentage [n(%)]**	**Counts [mean(sd)]**	**Percentage [n(%)]**	**Counts [mean(sd)]**	**Percentage [n(%)]**	**Counts [mean(sd)]**
F0	2936	2613 (89.0%)	2.7 (1.9)	3.0 (2.4)	1220 (46.7%)	0.6 (0.7)	2107 (80.6%)	1.3 (0.9)	1749 (66.9%)	1.1 (1.0)
F1	2936	2714 (92.4%)	3.2 (2.1)	3.6 (2.7)	1416 (52.2%)	0.7 (0.8)	2293 (84.5%)	1.5 (0.9)	1897 (69.9%)	1.2 (1.1)
F2	1621	1536 (94.8%)	3.4 (2.1)	3.9 (2.8)	850 (55.3%)	0.8 (0.8)	1323 (86.1%)	1.5 (0.9)	1098 (71.5%)	1.3 (1.1)
F3	1059	1021 (96.4%)	3.9 (2.3)	4.6 (3.1)	646 (63.3%)	0.9 (0.9)	912 (89.3%)	1.7 (1.0)	754 (73.8%)	1.4 (1.2)
F4	669	647 (96.7%)	4.2 (2.3)	5.0 (3.3)	423 (65.4%)	1.0 (0.9)	598 (92.4%)	1.9 (1.0)	491 (75.9%)	1.5 (1.2)

F0 represents the baseline (the wave in which participants first reported having diabetes), while F1 to F4 represent subsequent follow-up waves；The values for "Numbers of comorbidities", “CCI of comorbidities”, “TCC counts”, “NCC counts” and “DC counts” were calculated excluding diabetes.
